# A Cold-Induced LEA3 Protein, DohD, Confers Cryoprotective Protection Against Low-Temperature Stress in *Deinococcus radiodurans*

**DOI:** 10.3390/ijms26083511

**Published:** 2025-04-09

**Authors:** Wenxiu Wang, Zhi Qi, Chunxia Yan, Zhengfu Zhou, Jin Wang

**Affiliations:** 1 National Key Laboratory of Agricultural Microbiology, Biotechnology Research Institute, Chinese Academy of Agricultural Sciences, Beijing 100081, China; 2 Key Laboratory of Forage and Endemic Crop Biology, Ministry of Education, School of Life Science, Inner Mongolia University, Hohhot 010021, China

**Keywords:** *Deinococcus radiodurans*, low-temperature stress, DohD, cold-induced protein, LEA3

## Abstract

*Deinococcus radiodurans* is a remarkably unique microorganism, exhibiting extraordinary tolerance to extreme conditions such as ionizing radiation, ultraviolet light, and desiccation. However, the response mechanisms of *D. radiodurans* under low-temperature stress remain largely unexplored and have yet to be fully elucidated. The DohD protein is a hydrophilic member of the late embryogenesis abundant 3 (LEA3) family of *D. radiodurans*, playing a pivotal role in abiotic stress adaptation. Bioinformatics analysis revealed that DohD contains tandem repeats and disordered domains, with a remarkably high α-helix content (91.41%). Furthermore, DohD exhibits extremely low homology with other proteins, highlighting its uniqueness to *D. radiodurans*. Under low-temperature stress (15 °C), the expression of *dohD* was significantly upregulated (5-fold), regulated by a dual mechanism involving positive control by DrRRA and negative regulation by Csp. Circular dichroism spectroscopy unveiled temperature-dependent structural plasticity: as the temperature increased from 0 °C to 50° C, the α-helix content decreased from 23.5% to 18.7%, while the antiparallel β-sheet content increased from 31.3% to 50.8%. This suggests an α-helix to β-sheet interconversion mechanism as a strategy for thermal adaptation. Additionally, deletion of *dohD* impaired the tolerance of *D. radiodurans* to cold, desiccation, oxidative, and high-salt stresses, accompanied by the reduced activities of antioxidant enzymes (SOD, CAT, POD) and the downregulation of related gene expression. This study elucidates the multifunctional role of DohD in stress resistance through structural dynamics, transcriptional regulation, and redox homeostasis, providing valuable insights into the adaptation mechanisms of extremophiles.

## 1. Introduction

Cold-induced proteins (CIPs), a class of biomolecules uniquely evolved to maintain structural integrity and catalytic efficiency under low-temperature stress, serve as critical mediators of cold adaptation across diverse organisms. As pivotal regulators of cellular homeostasis, metabolic reprogramming, and ecological resilience, CIPs play essential roles in enabling organisms to thrive in thermally challenging environments [1,2,3]. CIPs are broadly categorized into functional subgroups based on their mechanisms of action. The first class includes chaperone-like proteases, exemplified by the PSME-3 activator in Caenorhabditis elegans, which extends the lifespan at 15 °C by enhancing the proteolytic degradation of misfolded proteins—a mechanism conserved in humans to mitigate neurodegenerative protein aggregation [4]. A second category comprises cryoprotective agents, such as ice-binding proteins, which inhibit ice crystal growth in frozen food systems, preserving macromolecular stability and improving post-thawing quality [5]. CIPs are further stratified into photosynthetic regulators, which sustain carbon fixation under cold stress by modulating Calvin cycle enzymes [6,7], and metabolic adjustors, which rewire nitrogen assimilation pathways through the dynamic expression of amino acid synthases [8]. Emerging evidence also highlights a distinct subclass of membrane-stabilizing CIPs that enhance lipid bilayer fluidity and cold-responsive signaling, crucial for maintaining cellular integrity [9]. Functional studies reveal CIPs as molecular “Swiss Army knives”, integrating structural plasticity with context-dependent activity. Their cold-adaptive traits—such as flexible catalytic domains, increased surface hydrophobicity, and cold-inducible post-translational modifications—enable enzymatic function under conditions of reduced kinetic energy [2,3].

In extreme environments, diverse microbial communities thrive, many of which possess cold-adapted proteins to cope with low-temperature conditions [10]. These specialized proteins play critical roles in maintaining cellular integrity by regulating membrane fluidity and permeability, while also facilitating essential metabolic processes, including methane oxidation and sulfate reduction [11,12]. Among these extremophiles, *D. radiodurans* stands out for its remarkable resilience to multiple environmental stressors, including high levels of ionizing radiation, ultraviolet exposure, desiccation, chemical toxicity, and extreme temperature fluctuations [13,14,15,16]; late embryogenesis abundant (LEA) proteins play a pivotal role in alleviating low-temperature-induced damage [17,18]. Characterized by their exceptional hydrophilicity and desiccation tolerance, these proteins have been shown to protect human oocytes during vitrification preservation through mechanisms involving the suppression of FOS gene expression and the modulation of oxidative stress pathways [19]. Notably, *D. radiodurans* contains three desiccation stress-related proteins (DR1172, DR1372, and DR0105) that exhibit homology to LEA proteins [20,21,22].

Our research focuses on the DR0105 protein, which is predicted to contain a unique intrinsically disordered domain designated as DohD (*D. radiodurans* orphan genes hydrophilic intrinsically disordered protein). Late embryogenesis abundant (LEA) proteins have been extensively characterized as stress protectants in plants and certain extremophilic organisms, yet their evolutionary mechanisms and functional diversification in bacterial systems remain poorly understood. Specifically, while the cold adaptation strategies of *D. radiodurans* have been well documented, the molecular mediators enabling its enzymatic machinery to maintain functionality under suboptimal temperatures have not been systematically investigated. This study aims to address two critical knowledge gaps: the evolutionary trajectory of LEA-like proteins in radiation-resistant bacteria, and the specific mechanisms through which these proteins confer cryoprotection. By integrating phylogenetic analysis with functional characterization of DohD—a newly identified cold-induced protein—we seek to establish a mechanistic framework explaining how structural adaptations in bacterial LEA homologs contribute to extreme environmental resilience.

## 2. Results

### 2.1. DohD Is a Hydrophilic LEA3 Protein Containing a Disordered Domain

The *dohD* gene is situated on chromosome I of *D. radiodurans*, spanning a total length of 492 base pairs (bp). The encoded DohD protein comprises 163 amino acids and features five tandem repeats, each consisting of 16 amino acids (Table 1). Structural analysis reveals that these repeats share significant similarity with the characteristic 11-amino-acid tandem repeats commonly observed in the LEA3 protein family.

**Figure 1:**
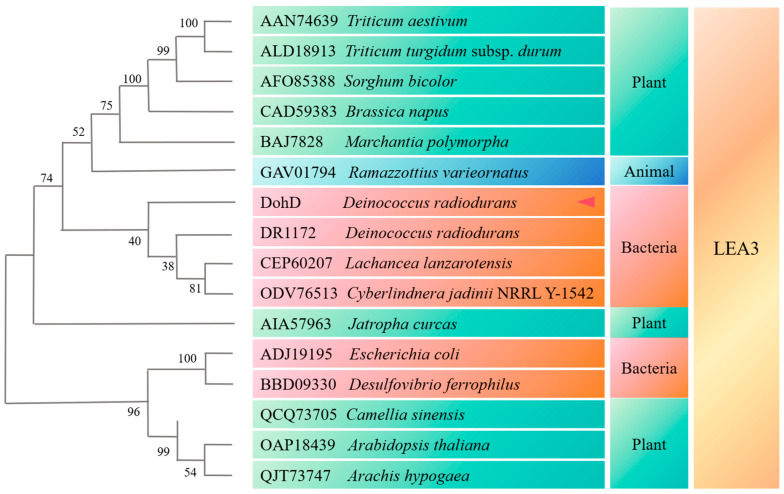
Phylogenetic analysis of DohD protein homologs. The evolutionary relationships were reconstructed using the neighbor-joining method implemented in MEGA 11.0 software, with branch lengths representing evolutionary distances between sequences. 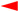: The target protein of this study.

Phylogenetic analysis demonstrated that the DohD protein exhibits the highest sequence similarity to a homologous protein from *Lachancea lanzarotensis* (Figure 1). However, comprehensive sequence alignment and comparative analysis revealed significant divergences between these proteins. Based on these findings, we propose that the DohD protein represents a unique functional protein specifically evolved in *D. radiodurans*.

**Figure 2:**
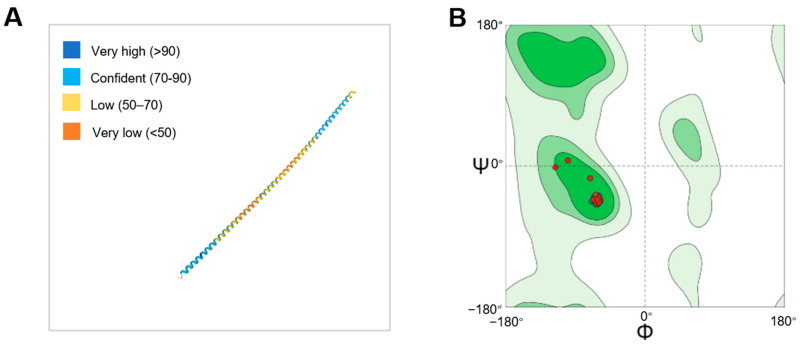
Structural analysis of the DohD protein. (**A**) Homology modeling of the predicted DohD protein structure; (**B**) Ramachandran plot of the DohD protein structure.

To further investigate the functional role of the DohD protein in low-temperature stress resistance, we conducted a comprehensive structural analysis. Secondary structure prediction revealed that 99% of the DohD protein adopts an α-helical conformation (Figure 2A). This predominant α-helical structure may play a crucial role in maintaining the structural stability of RNA or single-stranded DNA under low-temperature conditions. Specifically, the α-helical structure could prevent the formation of detrimental secondary structures in nucleic acids, thereby ensuring the proper progression of essential biological processes in *D. radiodurans* during cold stress.

Analysis of the Ramachandran plot for the DohD protein revealed that the dihedral angle distribution of φ (phi) and ψ (psi) indicates that most residues reside within the allowed regions, corresponding to secondary structures such as α-helices and β-sheets, which suggests high overall conformational stability (Figure 2B). Notably, some residues exhibit φ or ψ angles approaching 180°, likely reflecting extended conformations characteristic of β-sheets. This structural feature aligns with the β-barrel folding motif observed in typical cold-shock proteins, such as members of the Csp family, implying that DohD may maintain functional activity at low temperatures through a rigid β-sheet framework. In summary, the structural stability of DohD is closely associated with its low-temperature stress resistance, and the features of its Ramachandran plot further support its potential cold adaptation mechanism.

### 2.2. Low-Temperature Stress Induces DohD Expression

**Figure 3:**
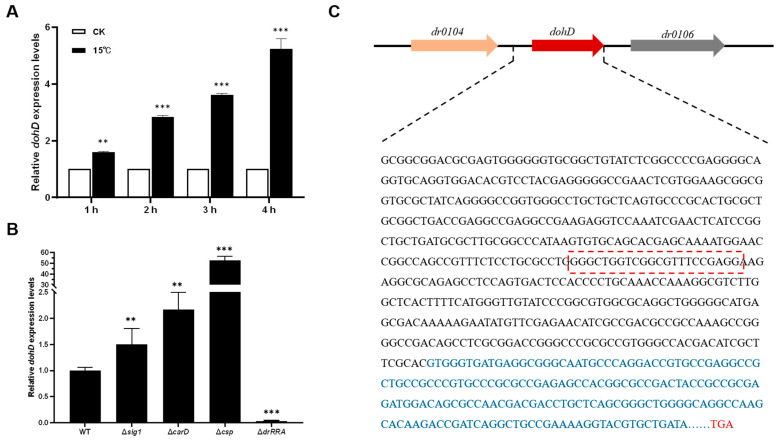
Transcriptional analysis of *dohD* in *D. radiodurans*. (**A**) Relative expression levels of *dohD* at 15 °C over different time intervals. (**B**) Relative expression levels of *dohD* in different mutant strains under 15 °C stress conditions. (**C**) Structural analysis of the *dohD* gene promoter in *D. radiodurans*. The sequence highlighted in the red box represents the predicted binding site for transcription factors DrRRA and Csp. The termination codon of the *dohD* gene is indicated in bold red letter. The *dohD* gene sequence is shown in blue and the *dohD* gene promoter region is shown in black. Statistical significance was determined using one-way ANOVA, followed by Dunnett’s multiple comparison test. A probability value of *p* ≤ 0.05 was considered statistically significant. Data are presented as means ± SEM. **: *p* ≤ 0.01; ***: *p* ≤ 0.001.

To investigate the transcriptional expression of the orphan gene *dohD* in *Deinococcus radiodurans* under abiotic stress conditions, wild-type *D. radiodurans* cells in the early logarithmic growth phase were exposed to normal and low-temperature treatments. The expression level of the *dohD* gene was quantified using qRT-PCR (Figure 3A). The results revealed that the most significant induction occurred after 4 h of stress treatment at 15 °C, with the expression of the *dohD* gene upregulated by approximately 5-fold. At other time points, the expression of the *dohD* gene also exhibited an increase. These findings suggest that the *dohD* gene can rapidly respond to low-temperature stress and may play a critical role in the low-temperature stress resistance mechanism of *D. radiodurans*.

Sig1, CarD, Csp, and DrRRA are key transcriptional regulators in *D. radiodurans*, and exert their effects through stress adaptation mechanisms such as oxidative stress response (Sig1, CarD), cold adaptation (Csp), ionizing radiation, and osmotic pressure adaptation (DrRRA), respectively. Therefore, they were selected as candidates to verify the upstream regulatory factors of the *dohD* gene. To elucidate the regulatory pathway of the DohD protein under low-temperature conditions, the expression of *dohD* was analyzed in the deletion mutants Δ*sig1*, Δ*carD*, Δ*csp*, and Δ*drRRA* at 15 °C using qRT-PCR (Figure 3B). Notably, *dohD* expression displayed contrasting regulatory patterns in Δ*csp* and Δ*drRRA* mutants: the deletion of *csp* resulted in a 53-fold upregulation of *dohD*, while the knockout of *drRRA* led to a 70-fold downregulation. In contrast, Δ*sig1* and Δ*carD* exhibited no significant changes in *dohD* expression, indicating their non-involvement in its regulation. These findings reveal a dual regulatory mechanism in which *dohD* is positively controlled by DrRRA and negatively regulated by Csp, establishing a balanced transcriptional network under cold stress.

To determine whether DrRRA and Csp directly regulate the *dohD* gene, this study examined the transcription factor binding sites within the promoter region of *dohD*. As shown in Figure 3C, the promoter region of *dohD* in *D. radiodurans* contains a conserved sequence motif, GGGCTGGTCGGCGTTTCCGAGG, which forms an inverted repeat interspersed with non-conserved bases. This sequence motif is likely to represent the core recognition site for the binding of transcription factors DrRRA and Csp to the promoter.

### 2.3. The Role of Secondary Structural Changes in the DohD Protein in Low-Temperature Stress Resistance

**Figure 4:**
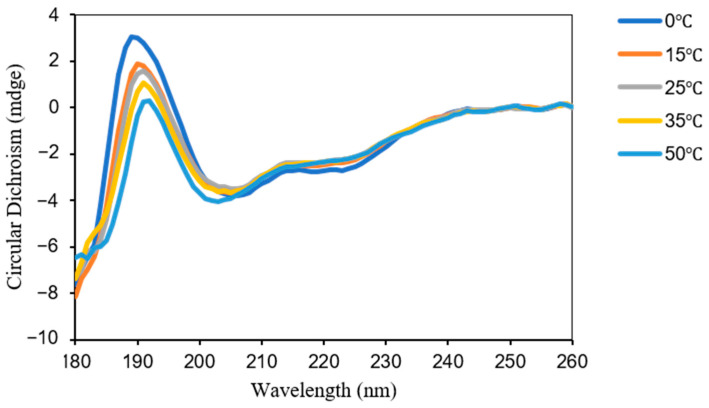
Secondary structure transition of DohD monitored by circular dichroism spectroscopy at different temperatures.

To further investigate the structural changes of the DohD protein under low-temperature conditions, this study systematically analyzed the dynamic alterations in its secondary structure across a temperature gradient ranging from 0 °C to 50 °C using circular dichroism spectroscopy. These analyses revealed the low-temperature adaptive structural characteristics of the protein. Experimental data demonstrated that temperature variations significantly influence the secondary structure composition of the DohD protein [23]. Specifically, as the temperature increased from 0 °C to 50 °C, the α-helix content (Helix%) gradually decreased (23.5% → 18.7%), while the antiparallel β-sheet content (Antiparallel%) exhibited an inverse growth trend (31.3% → 50.8%). In contrast, the fluctuations in β-turn (Turn%) and random coil (Random coil%) content remained within a range of 3.2% (Figure 4). This pattern of structural reorganization suggests that temperature regulation may be mediated through an “α-helix ↔ β-sheet” interconversion mechanism.

Under low-temperature conditions (0 °C), the DohD protein exhibits characteristic structural stability, marked by a higher proportion of α-helix (23.5%) and a lower proportion of antiparallel β-sheet (31.3%) content, which together establish a structural steady-state equilibrium. The α-helix, as a rigid structural element, likely maintains its abundance through a stable hydrogen bond network, thereby enhancing the protein’s structural order. This feature aligns closely with the structural strategies employed by psychrophilic proteins to preserve functional activity at low temperatures. When the temperature increases to 50 °C, the significant enrichment of antiparallel β-sheet (50.8%) suggests a new structural stabilization mechanism, i.e., the formation of an interlocking network through the expansion of the β-sheet structure, compensating for the loss of rigidity resulting from the unwinding of the α-helices (Table 2).

Notably, the proportions of β-turn and random coil structures remain relatively stable across the temperature range (with a fluctuation of 3.2%), suggesting that these structural elements may function as molecular “hinges”, facilitating localized conformational adjustments rather than driving the overall structural reorganization. This observation aligns with established theories of protein conformational dynamics, which propose that flexible regions enable the overall structure to adapt to environmental perturbations through limited conformational changes.

This study reveals that the DohD protein possesses a unique temperature-responsive structural regulation mechanism: it maintains a high α-helix content to ensure structural rigidity at low temperatures, while initiating β-sheet reorganization to achieve structural restabilization at elevated temperatures. This dual-mode regulatory strategy likely underpins its molecular basis for low-temperature stress resistance.

**Figure 5:**
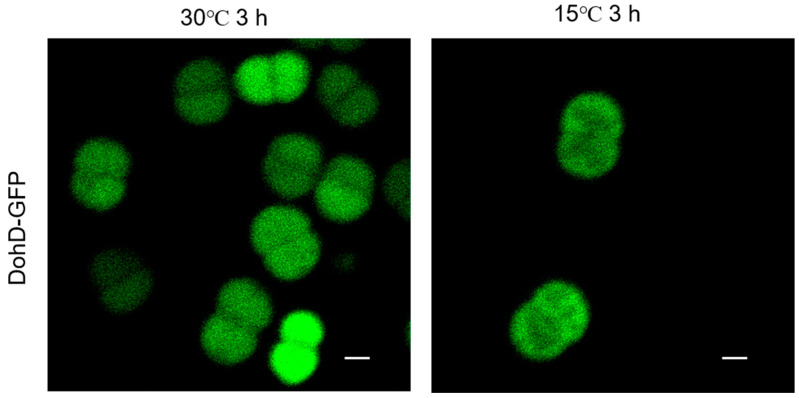
Subcellular localization of DohD protein under normal (30 °C) and low-temperature stress (15 °C) conditions. Green fluorescence represents DohD fused with GFP. Scale bar: 1 µm.

Subcellular localization analysis using laser confocal microscopy demonstrated the temperature-dependent redistribution of DohD. At 30 °C, DohD exhibited a uniform cytoplasmic distribution (Figure 5, left), indicating its potential role in fundamental cellular processes. Under cold stress conditions (15 °C), DohD underwent significant relocalization, characterized by the formation of dense membrane-associated clusters, which predominated over the diffusely distributed cytoplasmic pools (Figure 5, right). This membrane enrichment suggests potential interactions with stress-sensing or signaling components at the membrane interface during cold adaptation.

### 2.4. Deletion of dohD Decreases Abiotic Stress Tolerances of D. radiodurans

**Figure 6:**
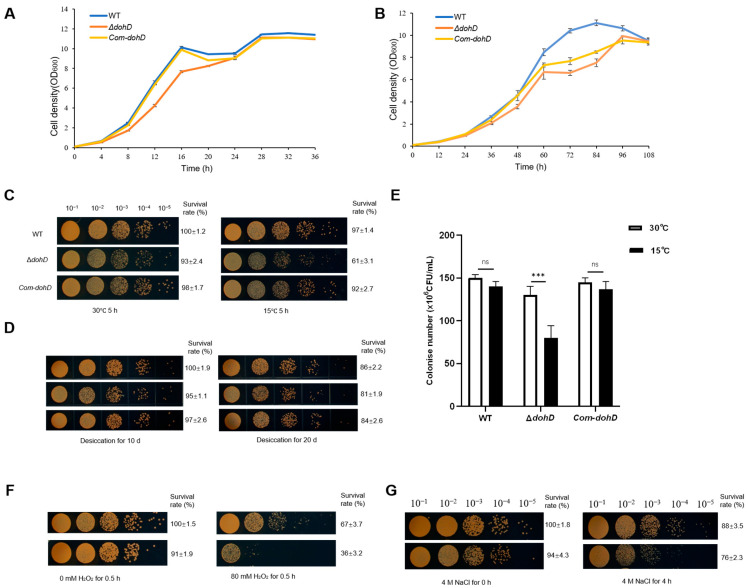
The deletion of the *dohD* gene reduced the low temperature and desiccation stress tolerance of *D. radiodurans*. (**A**) The growth curves of different *D. radiodurans* strains under normal culture temperature conditions. (**B**) The growth curves of different *D. radiodurans* strains under low temperature conditions. (**C**) Temperature sensitivity assays of different *D. radiodurans* strains. Spotted agar plates after temperature treatment and serial dilution: (left) 30 °C for 5 h.; (right) 15 °C for 5 h. (**D**) Desiccation sensitivity assays of different *D. radiodurans* strains. Spotted agar plates after desiccation treatment and serial dilution: (left) desiccation for 10 days; (right) desiccation for 20 days. (**E**) Statistics of colony number of different *D. radiodurans* strains at 15 °C for 5 h. One-way ANOVA and Dunnett’s multiple comparison test; a probability value of *p* ≤ 0.05 was considered significant. Data are presented as averages ± SEM. ***: *p* ≤ 0.001; ns: non-signifAicant. (**F**) Growth upon oxidative stress of *D. radiodurans* WT and mutant ΔdohD, under 80 mM H_2_O_2_ for 30 min and then spotted on TGY agar plates. (**G**) Growth upon high-salt stress of *D. radiodurans* WT and mutant Δ*dohD* under 4 M NaCl for 4 h and then spotted on TGY agar plates.

To determine whether the deletion of *dohD* affects the growth of *D. radiodurans* R1, growth curves were analyzed under standard culture conditions (30 °C, 220 rpm, TGY medium). As illustrated in Figure 6A, the wild-type strain (DR-WT), the *dohD* deletion mutant (∆*dohD*), and the complementation strain (com-*dohD*) displayed nearly identical S-shaped growth curves, with no significant differences in OD_600_ values during either the logarithmic or stationary phases. These results demonstrate that the deletion of *dohD* does not impair cellular proliferation under optimal growth conditions.

To investigate the role of *dohD* in abiotic stress resistance, we systematically assessed the viability of *D. radiodurans* strains under various stress conditions. Under cold stress (15 °C for 5 h), the colony-forming units (CFUs) of the *dohD* deletion mutant (∆*dohD*) decreased by one order of magnitude compared to the results for the wild-type strain (WT), accompanied by a 45% reduction in survival rate (Figure 6C,E). Time-dependent survival assays further revealed that after 60 h at 15 °C, ∆*dohD* exhibited a 25% growth decline relative to that of WT, while the complementation strain (com-*dohD*) showed partial recovery (15% decline, Figure 6B). These findings demonstrate that *dohD* plays a critical role in cold stress adaptation.

In addition to temperature sensitivity, the *dohD* deletion mutant (∆*dohD*) exhibited reduced tolerance to oxidative and high-salt stresses. The mutant strain demonstrated a significant decrease in antioxidant capacity (Figure 6F) and a two-order-of-magnitude reduction in survival under high-salt conditions compared to the results for the wild-type strain (WT, Figure 6G). Furthermore, under desiccation stress, ∆*dohD* displayed severely impaired growth and colony formation, while the complementation strain (com-*dohD*) partially restored the wild-type phenotype (Figure 6D).

Collectively, these findings demonstrate that the deletion of *dohD* significantly impairs the abiotic stress tolerance of *D. radiodurans*, encompassing cold, desiccation, oxidative, and high-salt stresses. The gene’s specific regulatory role in cold and desiccation stress responses, combined with its auxiliary functions in antioxidant defense and osmoregulation, underscores its multifunctional contribution to environmental adaptation. The partial phenotypic restoration observed in the complementation strain (com-*dohD*) further corroborates the functional specificity of *dohD* in the stress signaling pathways.

### 2.5. DohD Alleviates Cold-Induced Oxidative Damage by Maintaining Antioxidant Enzyme Activity

**Figure 7:**
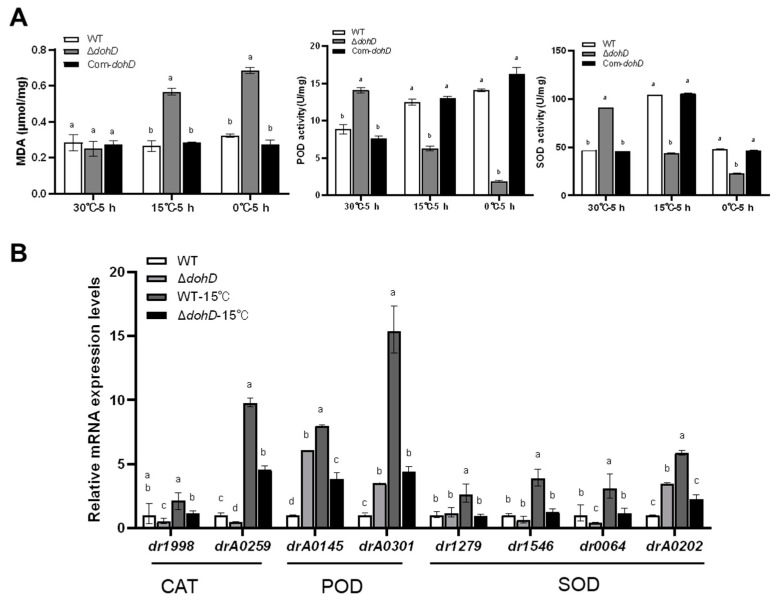
Expression and catalytic activities of ROS scavenging enzymes in *D. radiodruans* WT and Δ*dohD* mutant strains under low-temperature stress. (**A**) Effects of the *dohD* deletion on the enzyme activities after treatment with different temperatures for 5 h. (**B**) Effects of *dohD* deletion on the expression of antioxidant enzyme genes after treatment with different temperatures for 5 h. All experiments were performed in triplicate and are represented as mean ± standard deviation. Different letters indicate significant differences (*p* < 0.05).

Low-temperature stress induces the accumulation of reactive oxygen species (ROS) within cells, leading to lipid peroxidation of the cell membrane and the production of malondialdehyde (MDA). The elevated MDA content is widely used as a key indicator to evaluate the extent of cellular oxidative damage. In response to low-temperature stress, *D. radiodurans* maintains intracellular redox homeostasis by activating detoxifying enzymes such as superoxide dismutase (SOD) and catalase (CAT), thereby mitigating oxidative damage caused by low temperatures. To investigate the role of *dohD* in this process, we assessed the effects of *dohD* deletion on the expression and activity of ROS-scavenging enzymes, including CAT, peroxidase (POD), and SOD. Consistent with the low-temperature-sensitive phenotype of the Δ*dohD* mutant strain, the expression and activity of these ROS-scavenging enzymes were significantly lower than those in the wild-type (WT) strain after 5 h of low-temperature treatment (Figure 7A). Specifically, after 5 h at 15 °C, POD and SOD activities decreased by 2.2-fold and 2.1-fold, respectively, while at 0 °C, the reductions were 7.1-fold and 4.1-fold, respectively. Furthermore, the expression of genes encoding CAT (*dr1998* and *drA0259*), POD (*drA0145* and *drA0301*), and SOD (*dr1279*, *dr1546*, *dr0644*, and *drA0202*) was significantly downregulated in the Δ*dohD* mutant strain under low-temperature stress, consistent with the observed decline in antioxidant enzyme activities (Figure 7B). Collectively, these findings demonstrate that DohD is essential for the expression and activity of ROS-scavenging enzymes and likely plays a critical role in the extreme low-temperature tolerance of *D. radiodurans*.

### 2.6. DohD Enhances the Low-Temperature Stress Tolerance of E. coli

**Figure 8:**
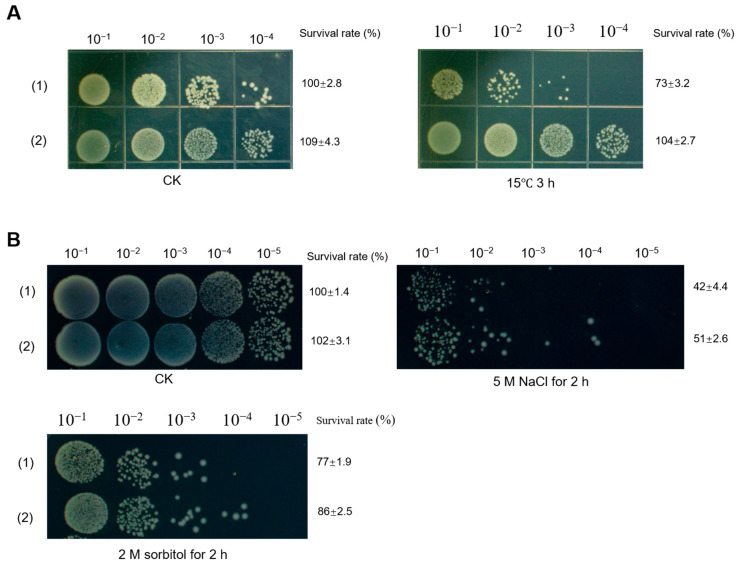
Phenotypes of DohD-expressing strain and control strain growth under various culture conditions. (**A**) Effect of culture at 15 °C on the growth of DohD-expressing strain and control strain. (**B**) Effect of culture at NaCl and sorbitol on the growth of DohD-expressing strain and control strain. (1) Control strain: pet28a/*E. coli* BL21(DE3); (2) DohD-expressing strain: pet28a+dohD/*E. coli* BL21(DE3).

To investigate the role of DohD in *Escherichia coli* tolerance to low-temperature stress, we performed bacterial survival assays. Cells grown to the logarithmic phase (OD600≈0.6) were exposed to 30 °C (control) or 15 °C (low-temperature stress) for 3 h. Serial dilutions (10^−4^) of the cultures were spotted (7 µL/dot) onto LB plates and incubated overnight at 37 °C. At 30 °C, all strains exhibited comparable growth, confirming the absence of baseline growth defects (Figure 8A, left). Notably, under 15 °C stress, the DohD-expressing strain demonstrated significantly enhanced survival compared to that of the control (Figure 8A, right), highlighting the critical role of DohD in low-temperature adaptation.

Remarkably, DohD also conferred cross-protection against osmotic stress. The DohD-expressing strain demonstrated significantly enhanced survival under high-salt conditions (Figure 8B), indicating its dual role in mitigating both thermal and osmotic stress. This multifunctional capability aligns with its stress-responsive localization dynamics, suggesting a coordinated mechanism for environmental adaptation.

## 3. Discussion

The extremophilic bacterium *D. radiodurans* employs a sophisticated arsenal of molecular strategies to withstand extreme environments. This study demonstrates that DohD, functioning as a molecular chaperone, enhances the strain’s resilience to stress. By integrating structural dynamics, transcriptional regulation, and antioxidant defense mechanisms, DohD orchestrates a multilayered adaptive response, providing novel insights into the stress adaptation strategies of *D. radiodurans*.

### 3.1. Structural Plasticity of the Cold-Inducible Protein DohD Drives Its Cold Adaptation Mechanism

Intrinsically disordered regions (IDRs) in stress-response proteins are increasingly recognized as mediators of structural flexibility [24], which may explain DohD’s ability to adopt multiple conformations (Figure S3). DohD exhibits pronounced transcriptional upregulation under cold stress (5-fold induction at 15 °C), regulated by the transcription factors DrRRA (positive regulation) and Csp (negative regulation). This induction aligns with functional necessity, as Δ*dohD* mutants displayed severe cold sensitivity, including reduced survival and impaired antioxidant enzyme activity. Central to its adaptive function is DohD’s structural plasticity, characterized by temperature-dependent α-helix to β-sheet interconversion. At low temperatures, the high α-helix content (23.5%) stabilizes RNA or single-stranded DNA through rigid hydrogen-bonded frameworks [25,26,27]. Conversely, under elevated temperatures (50 °C), the enrichment of antiparallel β-sheets (50.8%) suggests a compensatory mechanism to restore structural stability [28], highlighting DohD’s dual-mode conformational regulation. This demonstrates how bacterial LEA homologs achieve dual thermostability through conformational dynamics—a functional strategy unreported in eukaryotic systems. Notably, its conserved β-barrel folding pattern, reminiscent of that of cold-shock proteins (Csps) [29,30], underscores DohD’s evolutionary linkages to cold adaptation.

### 3.2. Regulatory Circuitry and Oxidative Defense

To elucidate how extremophiles coordinate stress-responsive gene expression, we identified a novel dual regulatory axis: DrRRA activates dohD (70-fold downregulation in Δ*drRRA*), while Csp suppresses it (53-fold upregulation in Δ*csp*) (Figure 3B). This counterbalanced system differs fundamentally from the single-factor regulation of plant LEA genes [31,32], suggesting evolutionary specialization in extremophiles. Crucially, this regulatory architecture directly links to DohD’s antioxidant function. Δ*dohD* mutants showed a 4.1-fold reduced SOD activity at 0 °C (Figure 7A), exceeding the 2.5-fold decline observed in Synechocystis LEA knockouts under similar conditions, underscoring DohD’s central role in redox homeostasis, a trait shared with extremophile-specific chaperones like *Deinococcus* DrwH [33], yet distinct in its transcriptional coordination. The above results quantitatively validate our hypothesis that bacterial LEA proteins integrate transcriptional control with redox homeostasis [34].

### 3.3. DohD Integrates Antioxidant Defense with Membrane Stabilization

**Figure 9:**
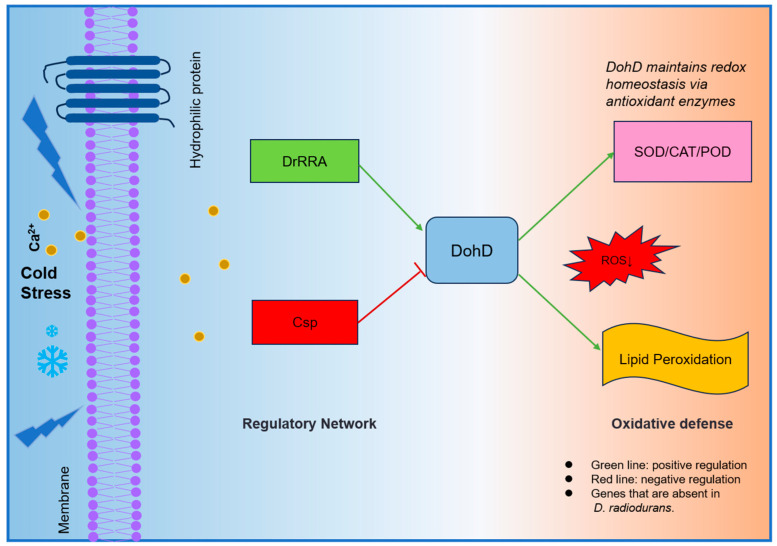
DohD regulatory network and low-temperature defense mechanism. This figure systematically illustrates the transcriptional regulatory pathway of the DohD protein under low-temperature stress and the oxidative defense mechanism it orchestrates.

DohD uniquely couples antioxidant defense (2.1-fold higher SOD/POD activities in WT vs. Δ*dohD*, Figure 7A) with membrane stabilization—a dual mechanism evidenced by its temperature-dependent relocalization (30 °C: cytoplasmic; 15 °C: membrane clusters, Figure 5). Cold stress induces ROS accumulation, posing a threat to cellular components through lipid peroxidation [35,36,37,38]. DohD mitigates this damage by sustaining the activities of antioxidant enzymes (SOD, CAT, POD) and the expression of their encoding genes. In Δ*dohD* mutants, SOD activity decreased by 4.1-fold at 0 °C (Figure 6A), which correlated with elevated MDA levels—a key marker of oxidative membrane damage [39,40]. This phenotype aligns with the role of Arabidopsis LEA proteins in scavenging ROS under dehydration stress [31,32,41], suggesting a conserved mechanism across diverse domains of life. In Figure 9, the regulatory mechanism of *D. radiodurans*’ tolerance to cold stress is summarized.

### 3.4. Cross-Species Functionality Highlights Evolutionary Conservation

Addressing the evolutionary conservation of cryoprotective mechanisms, heterologous DohD expression in *E. coli* conferred a 15% higher survival at 15 °C (Figure 8A)—a functionality absent in plant LEA3. Despite low sequence homology (Figure 1), this cross-kingdom compatibility suggests that α-helix clustering (Figure 2A) represents a universal structural motif for bacterial cryoprotection. Furthermore, the improved salt tolerance in *E. coli* (Figure 7B) underscores DohD’s multifunctionality, akin to the osmoprotective roles of *Bacillus* cold-shock proteins (CSPs). Such versatility aligns with the “moonlighting” capability of intrinsically disordered proteins, which adopt context-dependent conformations to fulfill diverse biological roles.

## 4. Materials and Methods

### 4.1. Bacterial Strains, Plasmids, and Media

The strains and plasmids used in this study are described in Supplementary Table S1. *D. radiodurans* WT and its derivatives were cultured aerobically at 30 °C in TGY medium (1% tryptone, 0.5% yeast extract, and 0.1% glucose) in the presence of antibiotics, as required. *E. coli* strains were grown in Luria–Bertani (LB) broth at 37 °C, with appropriate antibiotics. Solid media contained 1.5% agar.

### 4.2. Construction of DohD Deletion Mutants and Complementary Strains

The mutant strains were constructed according to the principle of homologous recombination (see Supplementary Figure S1), with primers designed according to the full sequence of the *dohD* gene; see Table S2 in the Supplementary Materials. Using the *D. radiodurans* R1 genome as a template, P1/P2 amplified the upstream fragment of *dohD* at 921 bp (U), and P5/P6 amplified the downstream fragment of *dohD* at 915 bp (D). Using the pKatAPH3 plasmid as a template, P3/P4 amplified the kanamycin resistance gene nptII fragment 970 bp (K). A homologous recombinase was used to catalyze the fusion of the three fragments, UKD, in a 1:1:1 molar ratio. P1/P6 primers were used as templates to amplify the fusion fragment UKD (3596 bp). Finally, the fusion fragments were imported into *D. radiodurans* R1, according to the experimental method previously reported [42]. Single colonies were screened for kanamycin resistance (10 µg/mL). The results confirmed that the nptII gene was inserted in the correct position, replacing the *dohD* gene. The obtained *dohD* deletion mutant was named ∆*dohD* and was used for further studies. The *dohD* gene was inserted between the nucleic acid endonuclease BamH I and Spe I sites of the pRADZ3 plasmid, and the recombinant plasmid was then transformed into the ∆*dohD* mutant strain for the construction of the complementary strain com-*dohD*.

### 4.3. Bioinformatics Analysis

Bioinformatics analyses were conducted on the DohD protein using various tools. The Kyte–Doolittle hydropathy prediction website (https://www.novoprolabs.com/tools/protein-hydropathy; accessed on 15 March 2023) was employed to analyze its hydrophobicity. The VSL2 website (http://www.pondr.com/; accessed on 10 August 2023) was used to predict the ordered and disordered regions. Transmembrane features were investigated using the TMHMM (transmembrane hidden Markov model) website (https://www.novopro.cn/tools/tmhmm.html; accessed on 17 April 2024). Phylogenetic analysis was carried out by aligning DohD sequences with homologs from different sources, including *Lachancea lanzarotensis*, archaeal CSPs, and bacterial Csp members, using sequence alignment tools and neighbor-joining methods. All protein sequences containing LEA3 domains were retrieved from the UniProt and NCBI databases using the keywords “LEA3” and “hydrophilic stress protein”. Phylogenetic analysis was performed for homologous proteins with sequence similarity ≥ 30% and coverage ≥ 70% (E value < 1 × 10^−5^) by BLASTP. Secondary structure prediction was performed with PSIPRED. Structural stability was assessed through Ramachandran plot analysis.

### 4.4. Real-Time Fluorescence Quantitative PCR for Gene Expression

*D. radiodurans* R1 organisms were collected under various abiotic stress conditions using the Ambion^®^RNA extraction kit from Life (Austin, TX, USA). The 16S gene was used as the internal reference, and qRT-PCR was performed for the *dohD*, *dr1998*, *drA0259*, *drA0145*, *drA0301*, *dr1279*, *dr1546*, *dr0644*, *drA0202*, *sig1*, *carD*, *csp,* and *drRRA* genes under different temperature conditions using the ^ΔΔ^Ct method. The qRT-PCR was carried out with an AB 7500 Real-Time PCR System, according to the manufacturer’s recommendations. The primer pairs used in this experiment are listed in Supplementary Table S2.

### 4.5. Activity Measurement of Major Antioxidant Enzymes

The *D. radiodurans* WT and Δ*dohD* mutant cells (OD600 ≈ 2, treated with 15 °C for 2 h and 30 °C for 2 h) were harvested by centrifugation, washed and resuspended with sterile PBS (pH 7.0), and disrupted on ice with an ultrasonicator. The debris was removed by centrifugation at 13,000× *g* at 4 °C for 20 min. Protein concentrations of the supernatants were determined by the Bradford method using BSA as the standard. The total SOD activity detection (WST-8 method) and lipid oxidation (MDA) detection kits were purchased from Shanghai Biyuntian Company (Shanghai, China), and the peroxidase (POD) detection kit was purchased from Nanjing, China. All experiments were repeated at least three times, independently.

### 4.6. Abiotic Stress-Resistance Assays

Low temperature resistance assay. A 1 mL sample of *D. radiodurans* R1 and its derivatives (with appropriate antibiotics) was incubated in TGY medium until the early stages of logarithmic growth (OD_600_ = 2) and cultured in a shaker at 15 °C and 30 °C (blank control group) for 1 to 5 h at 220 rpm, and a tube containing the sample was removed every 1 h and diluted to a final concentration of 10^−1^~10^−5^. An appropriate amount of the diluted sample was dropped onto the surface of the TGY solid medium and incubated in an incubator at 30 °C for 2–3 days to record the growth phenotype.

Desiccation resistance assay. A 1 mL sample of *D. radiodurans* R1 and its derivatives (with appropriate antibiotics) was incubated in TGY medium until the early stages of stable growth (OD_600_ = 8) and stored in a desiccator at room temperature for 60 days at relative humidity of less than 5%, following the reported method [33]. It was stored at room temperature in a desiccator at less than 5% relative humidity for 20 days. Samples were taken at 10-day intervals for cell recovery and diluted to a final concentration of 10^−1^~10^−5^. An appropriate amount of the diluted sample was dropped onto the surface of the TGY solid medium and incubated in an incubator at 30 °C for 2–3 days to record the growth phenotype.

### 4.7. DohD Expression and Purification

The *dohD* gene was amplified by PCR using genomic DNA from *D. radiodurans* R1 as the template, following the cloning strategy described in Reference [43]. The amplified fragment was directionally ligated into the pET28a expression vector via BamH I/Hind III double digestion to construct the recombinant plasmid, which was subsequently introduced into *E. coli* BL21(DE3) competent cells via electroporation. Protein expression was induced overnight at 16 °C with 0.5 mM isopropyl-β-D-thiogalactoside (IPTG). Bacterial cells were harvested under low-temperature conditions and lysed by ice-bath sonication. The supernatant and pellet fractions were separated by centrifugation and analyzed by SDS-PAGE using a Bio-Rad electrophoresis system from Bio-Rad Laboratories, Inc. Company (Hercules, CA, USA).

For protein purification, the nickel-affinity chromatography method, reported by Fredriksen [44], was modified as follows: The supernatant was filtered through a 0.22 μm membrane and loaded onto a His Trap HP nickel column (5 mL bed volume; GE Healthcare, Chicago, IL, USA) using an ÄKTA pure chromatography system. Fractions containing the target protein were pooled based on SDS-PAGE analysis, followed by desalting and buffer exchange using ultrafiltration concentrators. To prevent oxidative damage, the storage buffer was supplemented with 5 mM reduced glutathione and 2 mM dithiothreitol (DTT). The purified His-tagged DohD protein was stored at 4 °C for subsequent experiments, using the purification process visualized in Supplementary Figure S2.

### 4.8. Microscopy

For imaging of the *D. radiodurans* cells, a colony grown on a medium plate was inoculated into liquid medium and cultured overnight. The bacterial solution was cultured in an incubator at different temperatures. A total of 5 microliters of the bacterial solution was placed on a slide and covered with a coverslip. Imaging was performed on an AXR-NSPARC super-resolution confocal microscope(Carl Zeiss AG; Jena, Germany). GFP was excited at 488 nm and detected at 491–535 nm. Images were acquired with a ×100/1.46-NA oil objective.

## 5. Conclusions

DohD exemplifies *D. radiodurans*’ evolutionary ingenuity in merging structural disorder with functional precision. Through α/β structural switching, redox balancing, and dynamic localization, it orchestrates a multilayered defense against cold stress. Its integration into the DrRRA–Csp regulatory network further underscores the complexity of *D. radiodurans*’ adaptive mechanisms.

## Figures and Tables

**Table 1:** Repeated motifs of DohD protein.

Localization	Sequence
59–74	ADTQEAAQN⋯⋯AREKAQD
77–92	ANVHESAQD⋯⋯FRAGAQE
99–114	ADARDAAAQ⋯⋯ARDHAQN
117–136	TDVHEKAQD⋯⋯AHETAQN
150–163	ADVREI⋯⋯D⋯⋯RRSKNQD

**Table 2:** Percentage of helix, antiparallel, parallel, β-turn, and random coil structures in DohD protein obtained by far-UV CD spectrometry and calculated with CDNN software 2.1.

Treatment	Helix %	Antiparallel %	Parallel %	β-Turn %	Random Coil %
0 °C	23.5	31.3	9.1	21.1	25.7
15 °C	20.4	41.1	10.1	22.3	27.2
25 °C	20.1	42.1	10.3	22.4	27.9
35 °C	19.6	45.1	10.3	22.8	27.2
50 °C	18.7	50.8	10.3	23.6	26.3

## Data Availability

All data underlying the results are included as part of the published article and its Supplementary Materials.

## Supplementary Materials

The following supporting information can be downloaded at: https://www.mdpi.com/article/10.3390/ijms26083511/s1.
