# Supplementary material for: A Cold-Induced LEA3 Protein, DohD, Confers Cryoprotective Protection Against Low-Temperature Stress in Deinococcus radiodurans
Source: Int J Mol Sci. 2025 Apr 9;26(8):3511. doi: 10.3390/ijms26083511 (PMC12027078; doi:10.3390/ijms26083511)
Supplement: Supplementary file 1 [file ijms-26-03511-s001.zip › ijms-3545014-supplementary.pdf]

Supplementary Information

A Cold-Induced LEA3 Protein DohD Confers Cryoprotective Role in *Deinococcus radiodurans* Against Low-Temperature Stress

Wenxiu Wang [1,2], Xunqun Luo [3] , Zhi Qi[2] , Chunxia Yan[2] , Zhengfu Zhou[1,*] and Jin Wang [1,*]

Supplementary Figures

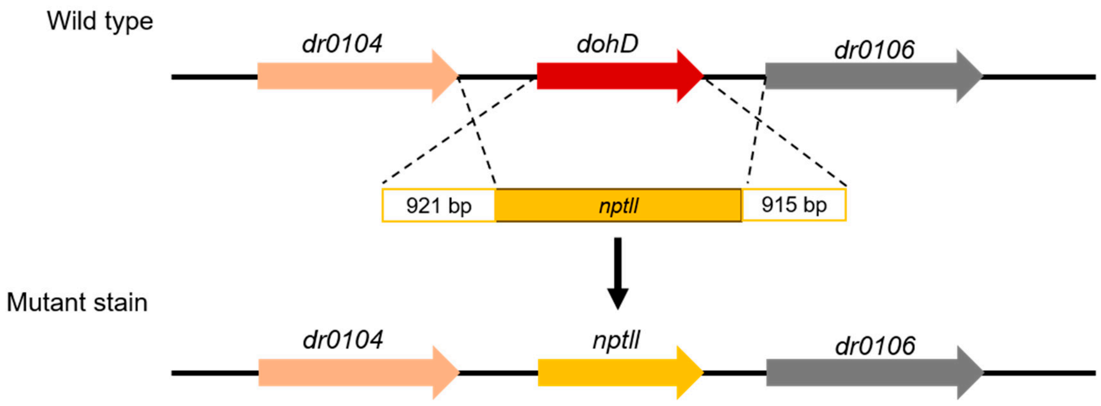

Figure S1. Construction and verification of the Δ*dohD* mutant. Schematic representation of the Δ*dohD* mutant generated by replacing the *dohD* region with the kanamycin resistance gene nptII (Kan^R).

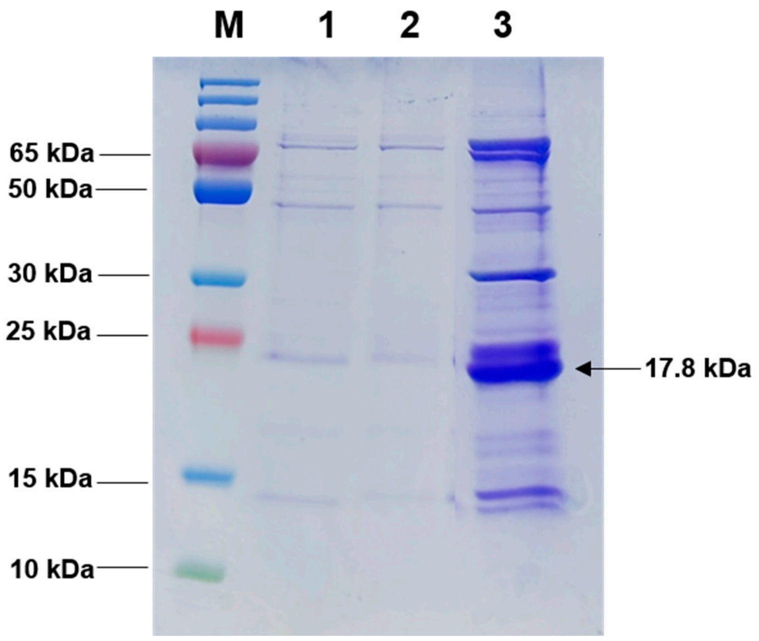

Figure S2. SDS-PAGE analysis of purified DohD. Cultures were incubated with 0.4 mM IPTG at 16°C overnight. Lane M, molecular weight standards (kDa); lane 1, supernatant after lysis; lane 2, flow through; and lane 3, elution with 120 mM imidazole.

To comprehensively characterize the physicochemical properties of the DohD protein, we utilized the Kyte-Doolittle hydropathy prediction algorithm to analyze its amino acid sequence. This computational tool calculates hydrophobicity indices for individual amino acids and generates quantitative output, where positive values correspond to hydrophobic regions and negative values indicate hydrophilic characteristics. Our analysis demonstrated that the DohD protein exhibits a consistently negative hydropathy profile throughout its sequence, confirming its classification as a highly hydrophilic protein (Figure S3A).

Furthermore, to characterize the structural organization of the DohD protein, we employed the VSL2 algorithm for intrinsic disorder prediction. This computational tool utilizes amino acid sequence information to identify ordered and disordered regions within protein structures. Our analysis revealed that the DohD protein predominantly exhibits intrinsic disorder characteristics, with no significant ordered structural domains detected throughout its sequence (Figure S3B).

To further characterize the structural properties of the DohD protein, we conducted transmembrane domain prediction using TMHMM (Transmembrane Hidden Markov Model). This computational algorithm, which employs a hidden Markov model framework, is specifically designed for the accurate identification of transmembrane helical regions in protein sequences. Our analysis revealed the absence of detectable transmembrane helical structures in the DohD protein sequence, suggesting that it is likely a non-transmembrane protein (Figure S3C). Furthermore, we performed secondary structure prediction using the PSIPRED algorithm, which indicated that 91.41% (149 residues) of the DohD protein sequence is predicted to adopt α-helical conformations (Figure S3D).

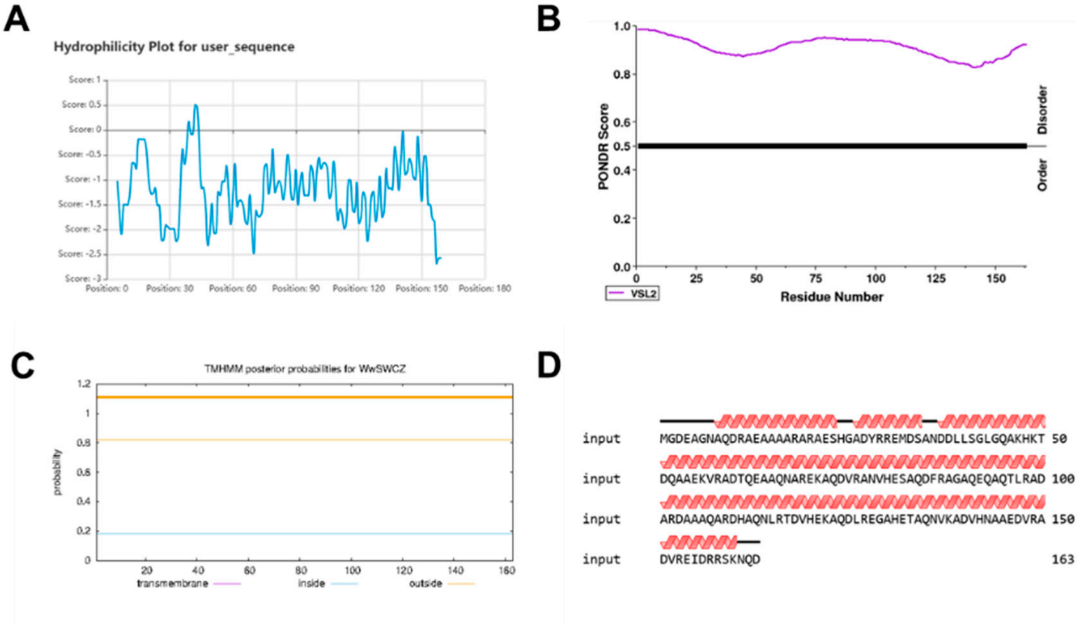

Figure S3. Bioinformatics analysis of DohD protein structural characteristics. (A) Hydropathy profile of the deduced DohD amino acid sequence generated using the Kyte-Doolittle algorithm. Hydrophobic regions are indicated by positive hydropathy index values (above the zero baseline). (B) Intrinsic disorder prediction of DohD protein using the VSL2 algorithm, with disordered regions identified by scores exceeding the threshold value (0.5). (C) Transmembrane domain prediction of DohD protein using TMHMM (Transmembrane Hidden Markov Model), demonstrating the absence of transmembrane helices. (D) Secondary structure prediction of DohD protein performed by PSIPRED, showing the distribution of α-helices, β-strands, and coil regions.

Supplementary Tables

Table S1. Bacterial strains and plasmids used in this study

**Strains and plasmids**	**Genotype or description**	**source**
D. radiodurans R1	Wild-type, served as the strain for generation of the mutants	Laboratory stock
Δ*dohD* mutant	*D. radiodurans* with genomic deletion of *dohD* gene *dohD* mutant with pRADZ3 shuttle plasmid introduced into its genome	This study
Com-*dohD*	Complementation of the *dohD* deletion in *D. radiodurans*, transformation of *dohD* mutant with pRADZ3 plasmid	This study
E. coli BL21	F^- *ompT* *hsdS*$_B$ (r_B^- m_B^-) *gal dcm* (DE3)	TransGen Biotech
BL21	The control strain harboring the empty vector pET28a, Kan^r	This study
BL21-*dohD*	The recombination BL21 strain containing pET-*dohD*, Kan^r	This study
pET28a (+)	Kan^r *oripBR322 lacI*^{q} *T7p*	Novagen
pET-*dohD*	pET28a carrying the wild-type *dohD* gene under the control of T7 promoter, Kan^r	This study
pRADZ3	The *E. coli*-*D. radiodurans* shuttle vector, Ap^r, Cm^r	Laboratory stock
pKatAPH3	To amplify the kanamycin resistance gene	Laboratory stock

Table S2. List of primers used in this study

Gene/Fragment name	Primers	Sequence (5′–3′)	Amplicon size (bp)
U	P1	ATCCTCACCCGCCGCCTACT	921
	P2	**GTTTTTCTAATCAGGATCCTCTAG**GTGCGAAGCGATGTCGTGGC	
nptII	P3	GCCACGACATCGCTTCGCAC**CTAGAGGATCCTGATTAGAAAAAC**	970
	P4	CCACTGGAAGGCGCAGGCGA**ACGGTATCGATAAGCTTGATAT**	
D	P5	**ATATCAAGCTTATCGATACCGT**TCGCCTGCGCCTTCCAGTGG	915
	P6	GCGGCAAACTGCGAAACCACCAC	
YZ-dohD	P7	GTGGGTGATGAGGCGGGCAAT	492
	P8	TCAGTCCTGGTTTTTGGAGCGG	
YZ-UKD	P9	TCGTGATGAAAGAGGTGGGT	3596
	P10	CGATAAAGGCGAGATTGGG	
drr06	RT16s-F	ATTCCTGGTGTAGCGGTG	146
	RT16s-R	CATCGTTTAGGGTGTGGAC	
dr0105 (dohD)	RT0105-F	GGGTGATGAGGCGGGCAAT	122
	RT0105-R	CGCTGAGCAGGTCGTCGTT	
dr1998 (cat)	RT1998-F	ACCAACATCCAGTCGCAG	167
	RT1998-R	CCTTCACCCTGGTCGTTG	
drA0259 (cat)	RTA0259-F	TGGGCAAGATGGTCCTCG	113
	RTA0259-R	CAGCGGGTCGTTGGTGA	
dr1279 (sod)	RT1279-F	TGCCCTACGCTTACGACG	159
	RT1279-R	CGAGCTGCTGAATGAGTTGT	
dr1546 (sod)	RT1546-F	GCACCGCCACCTTTAGC	108
	RT1546-R	TGCAGTTCGGGTTCTCG	
drA0202	RTA02	AACCGCAGTCTGGTCATCC	174

(sod)	02-F		
	RTA02 02-R	TTTCTTCGCGTCGTAGGC	
dr0644 (sod)	RT0644 -F	CTGGCTCTGATCGCTCTGC	172
	RT0644 -R	TGATGGTCGTGCGGGTC	
drA0145 (pod)	RTA01 45-F	TTCCCGAGTCGTTCAAAGTG	133
	RTA01 45-R	TCAGCGAAAATGGTCAGC	
drA0301 (pod)	RTA03 01-F	CGCTTTCAGGAGGTGTTCG	114
	RTA03 01-R	CGCCGAGTAGCGGTCATA	
